# Microbiota in Tumors: From Understanding to Application

**DOI:** 10.1002/advs.202200470

**Published:** 2022-05-23

**Authors:** Yifan Xie, Feng Xie, Xiaoxue Zhou, Lei Zhang, Bing Yang, Jun Huang, Fangwei Wang, Haiyan Yan, Linghui Zeng, Long Zhang, Fangfang Zhou

**Affiliations:** ^1^ School of Medicine Zhejiang University City College Suzhou 215123 P. R. China; ^2^ MOE Key Laboratory of Biosystems Homeostasis & Protection and Innovation Center for Cell Signaling Network Life Sciences Institute Zhejiang University Hangzhou Zhejiang 310058 P. R. China; ^3^ Institutes of Biology and Medical Science Soochow University Suzhou 215123 P. R. China; ^4^ Department of Orthopaedic Surgery Wenzhou The First Affiliated Hospital of Wenzhou Medical University Wenzhou 32500 P. R. China

**Keywords:** anti‐tumor therapy, intratumor microbiota, microbial community heterogeneity, omics technology, source of microbes, tumorigenesis

## Abstract

Microbes with complex functions have been found to be a potential component in tumor microenvironments. Due to their low biomass and other obstacles, intratumor microbiota is poorly understood. Mucosal sites and normal adjacent tissues are important sources of intratumor microbiota, while hematogenous spread also leads to the invasion of microbes. Intratumor microbiota affects the progression of tumors through several mechanisms, such as DNA damage, activation of oncogenic pathways, induction of immunosuppression, and metabolization of drugs. Notably, in different types of tumors, the composition and abundance of intratumor microbiota are highly heterogeneous and may play different roles in the progression of tumors. Because of the concern in this field, several techniques such as omics and immunological methods have been used to study intratumor microbiota. Here, recent progress in this field is reviewed, including the potential sources of intratumor microbiota, their functions and related mechanisms, and their heterogeneity. Techniques that can be used to study intratumor microbiota are also discussed. Moreover, research is summarized into the development of strategies that can be used in antitumor treatment and prospects for possible future research in this field.

## Graphical abstract

Intratumor microbiota is a hot spot in tumor research. In recent years, remarkable progress has been achieved in functions and application potential of intratumor microbiota. This review summarizes source, mechanisms, heterogeneity, and research techniques of intratumor microbiota, and discusses their potential for clinical translation to provide new insights into cancer treatment.

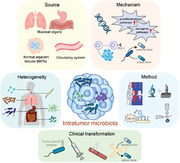

## 1 Background

**Figure 1:**
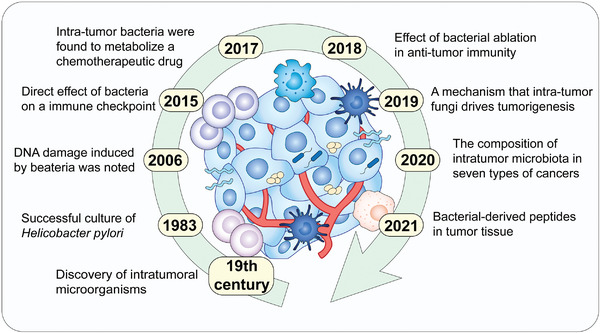
History of intratumor microbiota. Major breakthroughs from studies on intratumor microbiota including the discovery, mechanisms, and other achievements.

With the in‐depth study of host‐microbial interactions, the concept of intratumor microbiota present in tumor tissue was proposed. Intratumor microbes were first observed and described in the 19th century, but little progress in this field has been made in the following century.^[^ ^1^ ^]^ In recent years, with the development of detection technology and in‐depth understanding of tumor microenvironments (TMEs), increasing evidence has confirmed the existence of intratumor bacteria,^[^ ^2^, ^3^, ^4^ ^]^ which has led to more research in this field. Viruses have been associated with cancers such as human papillomavirus and Epstein‐Barr virus, and may induce the activation of oncogenesis directly;^[^ ^2^ ^]^ besides, bacteria, fungi, and other microbes present in tumors may also play complex roles in cancer development. In 1983, researchers cultured *Helicobacter pylori*, which is the main cause of gastric cancer.^[^ ^5^ ^]^ In 2006, DNA damage caused by bacteria was confirmed^[^ ^6^ ^]^ and more evidence has subsequently accumulated^[^ ^4^ ^]^ with much progress being made in this field (**Figure** **1**). In a recent study, peptides derived from intratumor bacteria were found to be present in tumor cells and elicit an immune response^[^ ^7^ ^]^ (Figure 1). In this review, we summarize the progress in the field of intratumor microbiota, including the source of intratumor microbes, the relationship between intratumor microbes and tumor development, the heterogeneity of intratumor microbiota in different types of tumors, and techniques to study intratumor microbiota.

## 2 Source of Intratumor Microbiota

**Figure 2:**
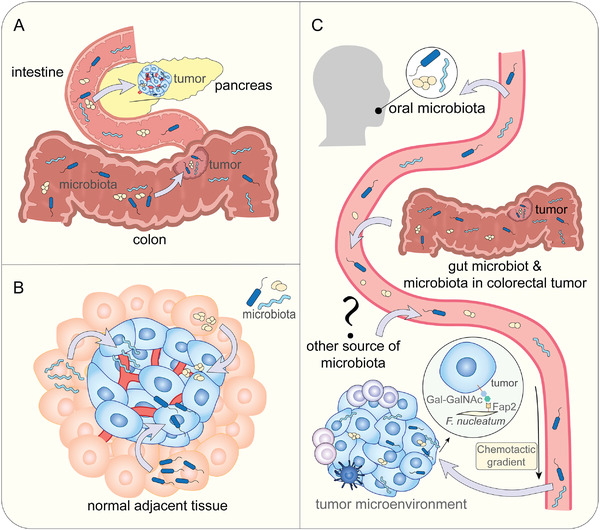
Sources of intratumor microbiota: A) Mucosal organs. Gut microbes disturb the mucosal barrier and enter tumor sites while intratumor bacteria of pancreatic cancer enter tumor sites through the pancreatic duct. B) NATs. NAT is a potential source of intratumor microbiota. C) Circulatory system. Intratumor microbes enter tumor sites from mouth, gut, tumors and other sites via hematogenous spread.

**Figure 3:**
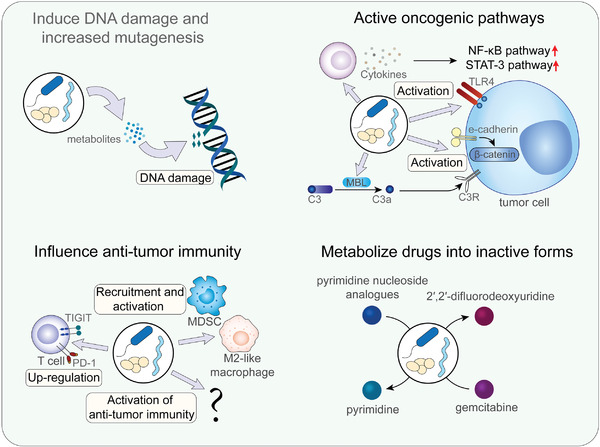
Mechanisms of intratumor microbiota affecting tumorigenesis and treatment. Two major mechanisms for inducing tumorigenesis include causing DNA damage and activating oncogenic pathways. . Intratumor microbiota also influence anti‐tumor immunity and play complex roles. Moreover, intratumor microbiota metabolize chemotherapeutic drugs and resulting in chemotherapy resistance.

Although intratumor microbes have received considerable attention, their sources are still unknown. In 2020, Walker et al. reviewed favorable conditions for bacterial colonization in tumors, including disorganized vasculature, hypoxic but nutrient‐rich microenvironments, and immunosuppression.^[^ ^8^ ^]^ In this review, we focus on three potential sources of intratumor microbes: 1) intratumor microbes originating from mucosal sites through mucosal barriers (**Figure** **2**A), 2) Intratumor microbes originating from normal adjacent tissues (NATs) (Figure 2B), and 3) Intratumor microbes which are the result of hematogenous spread (Figure 2C).

Abundant microbes exist in the mucosal organs of human bodies, such as the lung, gastrointestinal tract, and skin. Therefore, intratumor microbes are more likely to be found in cancers that arise from mucosal sites. Indeed, intratumor microbes have been discovered in lung cancer, colorectal cancer, pancreatic cancer, cervical cancer, and esophageal cancer.^[^ ^2^, ^4^ ^]^ In these cancers, tumorigenesis, as well as other causes of the breach of the body's mucosal barriers may provide access to the microbes resulting in them becoming intratumor microbiota capable of playing complex roles.^[^ ^9^ ^]^ In 2012, Tjalsma et al. proposed a bacterial driver–passenger model that partly explained the alteration of microbiota in the tumorigenesis of gastric cancers and colorectal cancers.^[^ ^10^ ^]^ In their model, “driver” bacteria like enterotoxigenic *Bacteroides fragilis* and *H. pylori* induce tumorigenesis and create conditions that allow “passenger” bacteria, which are normally opportunistic bacteria, to enter the TMEs and colonize there. The model may not explain the existence of intratumor microorganisms in several other cancers, such as lung cancer; however, it provides an indication that some microorganisms may play specific roles in facilitating the process of other microorganisms entering the TME. In addition, the breach of mucosal barriers caused by several factors may lead to the entrance of intra‐tumor microorganisms. It has been mentioned in several studies that intratumor bacteria found in pancreatic ductal adenocarcinoma transferred from the gut through the pancreatic duct, and the microenvironments of this adenocarcinoma may increase the susceptibility to bacterial translocation.^[^ ^11^, ^12^, ^13^ ^]^ Gut microbiota have been shown to decrease tumor burden in pancreatic cancers by influencing the composition of T cells and cytokines in a mouse model,^[^ ^14^ ^]^ which may indicate the invasion of gut bacteria into pancreatic tumors. More research is needed to determine the mechanisms by which microorganisms enter TMEs from mucosal organs, and this may improve the efficiency of therapies transferring bacteria to mucosal organs, especially fecal bacteria transplantation.

However, the idea that intratumor microbes come from mucosal sites through mucosal barriers cannot explain all the phenomena of intratumor microbes; some intratumor microorganisms are rare in mucosal organs where tumors occur, and microbes are also found in tumors that do not arise from mucosal sites, such as breast cancer, meaning there may be other sources of intratumor microbes. In a study in 2020, researchers proposed that intratumor bacteria may arise from NATs because the composition of the bacteria in tumor tissues and NATs have high similarity.^[^ ^15^ ^]^ With the development of related research, bacteria have been found in organs that are thought to be sterile.^[^ ^12^, ^16^ ^]^ In addition, intratumor microbes in some other tumors may also arise from NATs. Compared with NATs, specific characteristics, such as immunosuppressive and hypoxic microenvironments, enhance the microbes in tumor sites. Nevertheless, the sources of microorganisms in NATs are not fully understood. Another possible explanation for the similarity of the composition of microbiota in tumor sites and NATs, is that microorganisms in NATs originate from TMEs. Therefore, whether NATs are one of the sources of intratumor microbes is uncertain and more evidence is needed.

The circulatory system is another potential source of intratumor microbes. The chemotactic gradient of necrotic cellular debris may be the reason for the colonization by the intratumor microbiota.^[^ ^17^ ^]^ Systemic administration of bacteria has proven that microbes have the ability to colonize TMEs via hematogenous spread.^[^ ^18^ ^]^ Oral microbiota is an important source of intratumor microbiota. In 2016, researchers found that an oral bacterium, *Fusobacterium nucleatum*, could colonize colorectal cancer (CRC) via hematogenous spread. In this progression, lectin Fap2 plays an important role, attaching to Gal‐GalNAc expressed in CRC.^[^ ^19^ ^]^ In addition, oral microbiota has been found to be the source of pancreatic microbiota, which is related to pancreatic neoplasm.^[^ ^20^, ^21^, ^22^ ^]^ In 2018, Shively et al. reported that diet significantly affects the breast microbiome and thus influences the risk of breast cancer, which may suggest a close connection between gut microbiota and breast microbiota.^[^ ^23^ ^]^ Another recent study reported that gut fungi have a negative effect on the prognosis of breast cancer.^[^ ^24^ ^]^ Therefore, it can be implied that gut fungi and bacteria can invade the TMEs of breast tumors via hematogenous spread. In 2020, another group of researchers confirmed that *F. nucleatum* can also colonize breast cancer with the help of lectin Fap2.^[^ ^25^ ^]^ Notably, hematogenous spread of bacteria may lead to the formation of a “premetastatic niche.” In a study on colorectal cancer, researchers found that *Escherichia coli* in tumors can disrupt the gut vascular barrier and reach the liver via the hematogenous route. These intratumor bacteria boost the formation of a premetastatic niche.^[^ ^26^ ^]^ Considering the abundant and abnormal vascular systems in tumors, microorganisms from the mouth, gut, and other potential sites may be transported by the blood to tumor sites and enter tumors from damaged blood vessels.

With the deepening understanding of intratumor microbiota and several related studies, it is certain that microorganisms present in TMEs arise from multiple sources. In mucosal organs such as the gut, lung, and bladder, mucosal barrier damage caused by bacteria or other factors leads to the intrusion of opportunistic microorganisms that originally lived in these mucosal sites and play complex roles in TMEs. For microorganisms entering the blood circulation from multiple locations, the chemotactic gradient of necrotic cellular debris in tumors induces their transfer to tumor sites. Then, suitable environmental conditions in malformed blood vessels make it possible for them to colonize the TME. In NATs, microorganisms from blood vessels or mucosal organs may invade TMEs induced by oxygen and chemotactic gradients. Notably, microorganisms found in NATs or even in TMEs may be original species that massively proliferate due to the specific microenvironments created by tumorigenesis.

Importantly, with the source of intratumor microbes and related mechanisms being partly revealed, a deeper understanding of the intratumor microbiota may be used to improve the efficiency of associated therapeutic strategies. Considering the multi‐source features of intratumor microorganisms, comparing the composition of intratumor microbiota to microbiota in other sites in the body may help us to identify key microorganisms present in different tumors and provide new insights for cancer prevention. In addition, molecular mechanisms by which microorganisms invade TMEs are also an attractive topic for researchers while the understanding of them is still limited.

## 3 Mechanisms of Intratumor Microbiota Affecting Tumorigenesis and Treatment

Intratumor microbes play important roles in tumorigenesis and the treatment of cancers through multiple mechanisms.^[^ ^27^ ^]^ During tumorigenesis, some microbes may directly cause DNA damage, leading to the formation and progression of tumors. It has also been shown that some microbes induce the activation of proinflammatory responses and other oncogenic pathways. Intratumor microbes can also promote tumor progression by inducing immunosuppression and can affect the treatment of cancers in several ways. Some of these microbes have the ability to metabolize anti‐tumor drugs, while the alteration of anti‐tumor immunity may also influence the effect of cancer treatment (**Figure** **3**).

DNA damage leads to an increase in mutations, which may finally cause tumorigenesis. In several types of cancers, especially GI cancers, the microbiota is an important cause of DNA damage.^[^ ^9^, ^28^, ^29^ ^]^ Several studies have revealed that some members of *Enterobacteriaceae* produce colibactin, which causes DNA damage and induces tumorigenesis.^[^ ^6^, ^30^, ^31^, ^32^, ^33^ ^]^ A study conducted in 2020 further proved the mutagenic ability of colibactin, which is closely related to pathogenicity island *pks*.^[^ ^31^ ^]^ In this study, Pleguezuelos‐Manzano and colleagues observed a distinct mutational signature in organoids treated with genotoxic pks+ *E. coli*, while the mutational signature also existed in 5876 human cancer genomes. Another bacterial type that induces DNA damage is the enterotoxigenic *B. fragilis*, which can produce toxin. Dejea et al. reported the enrichment of enterotoxigenic *B. fragilis* and *E. coli* in the colonic mucosa of patients with familial adenomatous polyposis.^[^ ^34^ ^]^ Researchers have also demonstrated that there is more DNA damage in mice colonized with both bacteria. Another recent study also revealed that enterotoxigenic *B. fragilis* drove tumorigenesis, which further proved the carcinogenicity of this bacterium.^[^ ^35^ ^]^ Moreover, microbes can also induce oxidative/nitrosative DNA damage leading to tumorigenesis,^[^ ^36^ ^]^ although there is limited data on this effect.

The activation of oncogenic pathways is another effect of intratumor microbes. Researchers have revealed that certain intratumor microbes influence the production and secretion of cytokines, such as IL‐6 and TNF‐*α*. Several studies have shown that intratumor microbes may affect the production of cytokines, inducing a proinflammatory response, which then activated the NF‐*κ*B pathway or STAT3 pathway and promoted tumor progression.^[^ ^9^ ^]^ Triner et al. reported that intratumor bacteria induced the production of IL‐17, which promoted an influx of B cells, and the development of tumors.^[^ ^37^ ^]^ They also showed that neutrophils could restrict the intratumor microbiota and thus inhibit the development of tumors. However, few studies on the effect of intratumor microbes on cytokine production have been reported. Moreover, some members of Toll‐like receptors, such as TLR4 and TLR5, are closely associated with the interaction between tumors and microbes, including gut and intratumor microbes.^[^ ^38^, ^39^, ^40^ ^]^ A 2017 study showed that *F. nucleatum* activates the TLR4 signaling pathway, which affects the level of certain microRNAs, modulates autophagy, and finally causes resistance to chemotherapy.^[^ ^39^ ^]^ In 2021, Kong et al. reported that another pathway in *F. nucleatum*, induces colorectal cancer by increasing the levels of CYP2J2 and 12,13‐EpOME via activation of TLR4/Keap1/NRF2 signaling, leading to the invasion and metastasis of colorectal cancer.^[^ ^41^ ^]^ In addition, Hoste et al. reported that skin microbiota can also mediate proinflammatory responses via TLR‐5 signaling, thus affecting the progression of skin cancers.^[^ ^42^ ^]^ Wnt/*β*‐catenin signaling is another important oncogenic pathway that leads to the activation of several oncogenes. It has been proven that many kinds of microbes affect this pathway and induce tumorigenesis by activating *β*‐catenin directly or activating E‐cadherin.^[^ ^9^ ^]^ In addition, the complement system also plays an important role in tumorigenesis. In 2019, a study proved the carcinogenesis induced by fungi. The research found that certain fungi like *Malassezia* can bind mannose‐binding lectin (MBL) via their fungal wall glycans. MBL binding with fungi activates the complement cascade and therefore promotes the progression of PDA.^[^ ^43^ ^]^ Although there are several reports about the effect of intratumor microbiota on the activation of oncogenic pathways, most of these focus on GI cancers. Whether intratumor microbes play similar roles in other cancers, such as breast cancer and bone cancers, is uncertain.

Intratumor microbes also affect the immune microenvironment of tumors and therefore influence tumorigenesis and cancer treatment. Checkpoint blockade has received extensive attention as the most popular anti‐tumor strategy. Intratumor microbes may affect checkpoint proteins and affect immune microenvironments. Research has shown that Fap2 expressed by *F. nucleatum* binds to the checkpoint protein TIGIT directly and inhibits the anti‐tumor activity of NK cells as well as T cells.^[^ ^44^ ^]^ Another group of researchers observed upregulation of PD‐1 after bacterial ablation.^[^ ^13^ ^]^ Intratumor microbes can also affect the recruitment of immunosuppressive cells and bacteria have been shown to promote the recruitment of myeloid‐derived suppressor cells.^[^ ^13^, ^45^, ^46^, ^47^ ^]^ In 2018, Jin et al. reported an immune response associated with lung cancer induced by intratumor microbiota.^[^ ^48^ ^]^ These researchers showed that bacteria in cancer tissue induced the expansion of *γδ* T cells by activating the expression of IL‐1*β* and IL‐23. *γδ* T cells promote tumor progression by releasing IL‐17 and IL‐22 In 2020, researchers found that bacterial ablation promoted the differentiation of M1‐like macrophages and Th1 cells, indicating that bacterial ablation partly relieved immunosuppression in TMEs.^[^ ^13^ ^]^

Although intratumor microbiota may induce tumorigenesis in several ways, intratumor microbiota can inhibit the progression of tumors in some cases. A group of researchers discovered that the diversity of intratumor microbiota may influence the survival rate of patients with pancreatic cancer.^[^ ^49^ ^]^ They also reported that the composition of intratumor microbes in pancreatic cancer is highly associated with the composition of gut microbiota and that the alteration of gut microbiota influenced intratumor microbiota in pancreatic cancer. Kim et al. discovered the anti‐tumor effect of bacterial outer membrane vesicles, which induced the release of IFN‐*γ*,^[^ ^50^ ^]^ which indicates that intratumor microbiota may play complex roles in the occurrence and development of tumors. However, whether this mechanism is present in the TME is still unknown. Another possible mechanism is that intratumor microbiota promotes anti‐tumor immunity by producing special compounds, which have been proven in the research into gut microbes.^[^ ^51^, ^52^ ^]^ However, there is still no evidence that these compounds are released by intratumor microbes or that they improve anti‐tumor immunity.

In addition to the mechanisms mentioned above, intratumor microbes can also influence the effect of chemotherapy directly by metabolizing drugs into their inactive forms. In 2008, Balzarini et al. described the inactivation of pyrimidine nucleosides caused by certain microbes. In that report, researchers discovered that *Mycoplasma hyorhinis* can metabolize several pyrimidine nucleosides and influence their effects.^[^ ^53^ ^]^ Similarly, another study reported that gemcitabine, an important drug used in the chemotherapy of pancreatic cancers, can be metabolized into 2’,2’‐difluorodeoxyuridine, thereby losing its activation.^[^ ^54^ ^]^ Researchers have also found that metabolism is dependent on an enzyme expressed by intratumor microbes, mainly *Gammaproteobacteria*. Therefore, these discoveries remind us that we need to know more about the metabolic effects of intratumor microbes which can be used to develop specific strategies to treat cancers.

We have discovered several mechanisms by which intratumor microbes play complex or even contradictory roles. However, most of these mechanisms have only been proven in a limited number of cancers, and few studies have been successful in clinical trials. In such cases, more research is needed to clarify the mechanisms of the interaction between TMEs and intratumor microbiota.

## 4 Heterogeneity of Intratumor Microbiota in Different Tumors

TMEs are highly heterogeneous, and in different cancers, the composition and abundance of intratumoral microorganisms are also significantly different (**Table** **1**). The effects, therefore, of intratumor microbiota on different cancers may also be different. Notably, the composition and functions of the intratumor microbiota may vary among different subtypes of cancer. Moreover, little is known about the formation of microbial heterogeneity in different tumors and the relationship between the heterogeneity of TMEs and intratumor microbiota. Therefore, it is necessary to analyze the intratumor microbiota in different cancers and understand their roles in the progression of cancers.

**Table 1:** Microbial heterogeneity in different tumors

Tumor type	Microorganisms	Quantitative dynamics	Function
Lung cancer	*Thermus* ^[^ ^55^ ^]^	Increase	Related to advanced tumor
	*Legionella* ^[^ ^55^ ^]^		Related to tumor metastasis
	*Acidovorax* ^[^ ^56^ ^]^		Related to tumor high TP53 mutation
	*Streptococcus*, *Veillonella* ^[^ ^57^, ^58^ ^]^		Up‐regulation of ERK, PI3K and other pathway
	*Herbaspirillum*,*Sphingomonadaceae* ^[^ ^48^ ^]^		Activating *γδ* T cells
	*Aggregatibacter*, *Lactobacillus* ^[^ ^48^ ^]^	Decrease	
Esophageal adenocarcinoma	*Bacteroidetes*, *Firmicutes*, *Fusobacteria* ^[^ ^59^ ^]^	Increase	
	*F. nucleatum* ^[^ ^60^ ^]^		Inducing chemokine release
	*Proteobacteria* ^[^ ^59^ ^]^	Decrease	
Gastric cancer	*Peptostreptococcus stomatis, Streptococcus anginosus, Parvimonas micra, Slackia exigua, Dialister pneumosintes* ^[^ ^61^ ^]^	Increase	Related to tumor pregression
	*Actinobacteria, Firmicutes, non‐Helicobacter Proteobacteria* ^[^ ^62^ ^]^		
	*Bacteroidetes, Fusobacteria* ^[^ ^62^ ^]^	Decrease	
Colorectal cancer	*Alistipes,Bautia* ^[^ ^59^ ^]^	No significant difference	Related to better prognosis
	*Bacteroides, Parvimonas, Prevotella* ^[^ ^63^, ^64^ ^]^	Heterogeneous	Related to tumor progression
	*Fusobacterium* ^[^ ^59^, ^65^ ^]^	Increase	Related to tumor metastasis and progression
	*Escherichia coli*, *Bacteroides fragilis* ^[^ ^26^ ^]^		Inducing IL‐17 release and DNA damage
	*Bifidobacterium* ^[^ ^18^ ^]^		Activating STING pathway
	*Bacteroidetes*, *Bacteroideles* ^[^ ^59^ ^]^	Decrease	
Ovarian cancer	*Chlamydia*, *Mycoplasma*, *Acinetobacter*, *Brucella* ^[^ ^66^ ^]^	Increase	Direct or indirect tumorigenesis
Endometrial cancer	*Firmicutes, Spirochaetes, Actinobacteria, Bacteroidetes, Proteobacteria* ^[^ ^67^ ^]^		*A. vaginae* and *Porphyromonas sp*. are highly related to tumorigenesis
Pancreatic cancer	*Gammaproteobacteria* ^[^ ^54^ ^]^		Affecting anti‐tumor immunity and efficiency of chemotherapy
	*Malassezia globose* ^[^ ^43^ ^]^		Activating complement pathway and inducing tumorigenesis
	*Pseudoxanthomonas*, *Streptomyces* ^[^ ^49^ ^]^		Related to better prognosis
Pancreatic cyst	*Bacteroides*, *Escherichia*, *Acidaminococcus* ^[^ ^68^ ^]^	Increase	
	*Fusobacterium nucleatum*,*Granulicatella adiacens* ^[^ ^20^ ^]^	Increase	
Melanoma	*Acinetobacter, Actinomyces*, *Corynebacterium*, *Enterobacter*, *Streptococcus* ^[^ ^69^ ^]^		
Non‐melanoma skin cancer	*Staphylococcus aureus* ^[^ ^70^ ^]^	Increase	Related to carcinogenesis
	*Malassezia* ^[^ ^70^ ^]^	Decrease	
	*Staphylococcus epidermidis* ^[^ ^71^ ^]^		Producting 6‐N‐hydroxyaminopurine against tumor
Breast cancer	*Pseudomonas*, *Porphyromonas*, *Proteus*, *Azomonas* ^[^ ^72^ ^]^	Increase	
	*Enterotoxigenic Bacteroides fragilis* ^[^ ^73^ ^]^		Mediating *β*‐catenin and Notch1 axis and promoting tumor progression
	*Propionbacterium*, *Staphylococcus* ^[^ ^72^ ^]^	Decrease	
Head and neck cancer	*Parvimonas* ^[^ ^74^ ^]^	Increase	Related to tumor progression
	*Actinomyces* ^[^ ^74^ ^]^	Decrease	

**Figure 4:**
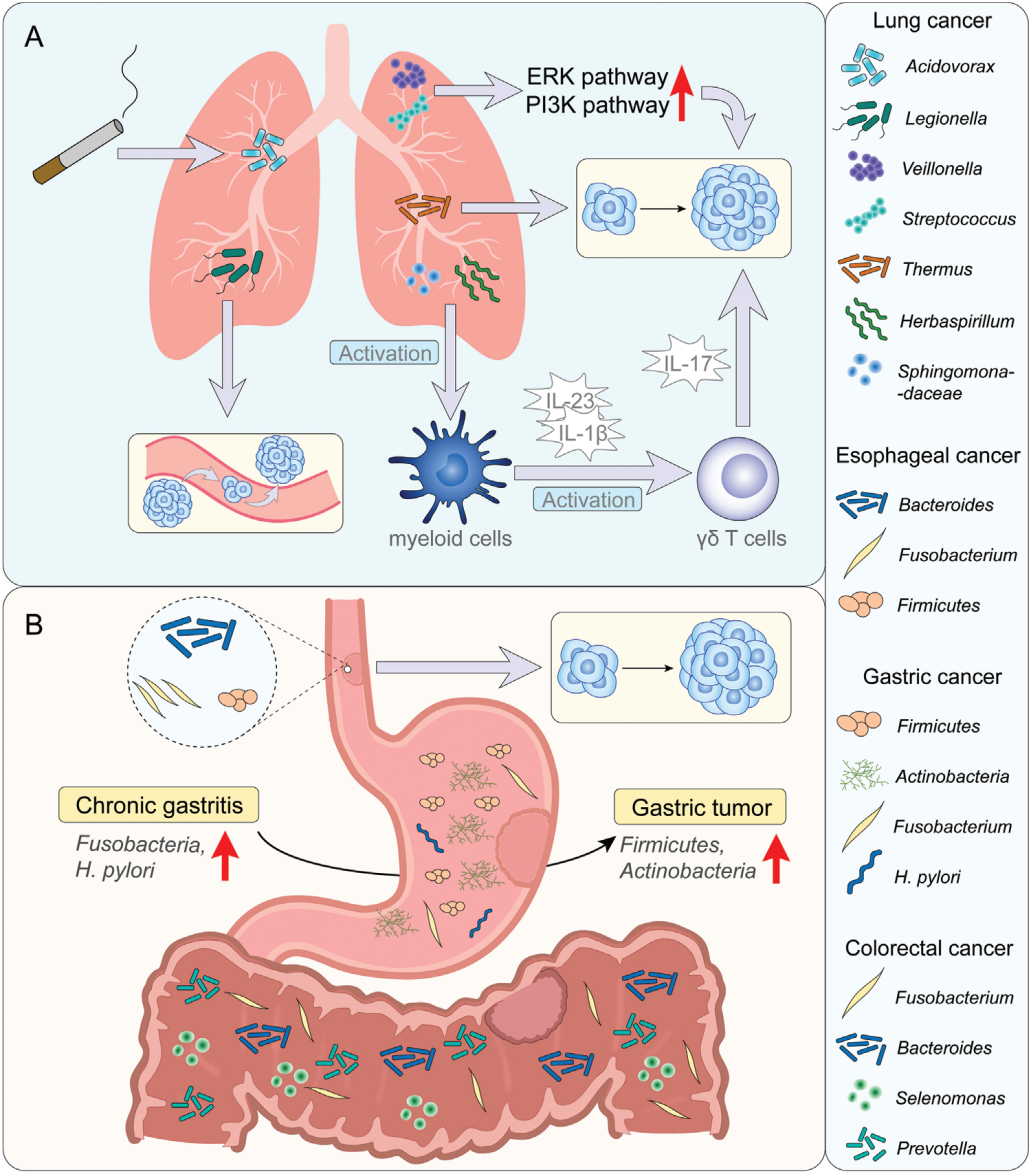
Heterogeneity of intratumor microbiota in different tumors A) Lung tumors. There are several bacteria that influence the progress and metastasis of lung cancers. These bacteria perform their roles using different pathways. B)Gastrointestinal tumors. The composition and function of microbiota in gastrointestinal tumors is complex. *Firmicutes*, *Selenomonas* and several other bacteria are closely related to the progression of tumors.

As a mucosal organ, the lungs are exposed to a rich microbiome (**Figure** **4**A).^[^ ^55^, ^75^, ^76^ ^]^ In lung cancer, the *α* diversity of microbiota decreases with the enrichment of certain bacteria.^[^ ^55^, ^56^ ^]^ Moreover, it is certain that the composition of lung microbiota is altered in lung cancers. Yu et al. showed that *Thermus* increased in patients with advanced tumors, while *Legionella* increased in patients with tumor metastasis.^[^ ^55^ ^]^ In addition, it has been revealed that smoking‐related metabolic pathways are enriched in lung cancer and certain groups of microbes, such as *Acidovorax*, increase in smokers,^[^ ^56^ ^]^ thus proving the relationship between microbiota and smoking. The richness of certain microbes in the lower airway may also induce lung cancer. It has been reported that the activation of the ERK, PI3K, and other pathways commonly found in patients with lung cancers is correlated with certain microbiota such as *Veillonella* and *Streptococcus*.^[^ ^57^, ^58^ ^]^ Notably, the alteration of the diversity and composition of microbiota in lung cancers is different in adenocarcinoma and squamous cell carcinoma,^[^ ^55^, ^56^ ^]^ which reminds us that the intratumor microbiota may be variable in different subtypes of cancer and may play complex roles. Microbiota also plays an important role in the treatment of lung cancers. It has been proven that the use of antibiotics has negative effects on immune checkpoint therapy in patients with lung cancer, although the analysis of intratumor microbiota is lacking.^[^ ^77^, ^78^ ^]^ Therefore, the combination of antibiotics, other bacterial therapies, and anti‐tumor immune therapies may have a better clinical effect.

Another type of cancer occurring at mucosal sites is gastrointestinal cancer, which is highly influenced by the microbiota (Figure 4B). A recent study analyzed the composition of intratumor microbes in four gastrointestinal cancers.^[^ ^59^ ^]^ In this study, researchers found that *Bacteroidetes*, *Firmicutes*, *Proteobacteria*, *Fusobacteria*, and *Actinobacteria* were the most abundant taxa at the phylum level in gastrointestinal cancers. In addition, the composition of bacteria in these cancers is significantly different from that in normal tissues.

In esophageal adenocarcinoma, the abundance of *Bacteroidetes*, *Firmicutes*, and *Fusobacteria* was increased compared with that in normal tissues, while the abundance of *Proteobacteria* decreased.^[^ ^59^ ^]^ Another study demonstrated an association between *F. nucleatum* and poor prognosis in esophageal cancers.^[^ ^60^ ^]^

In gastric cancer, the role of *H. pylori* in gastric carcinogenesis has been widely described,^[^ ^79^, ^80^, ^81^ ^]^ and is responsible for the alteration of microbiota in gastric cancers.^[^ ^82^ ^]^ The increase in non‐Helicobacter *Proteobacteria*, *Firmicutes*, and *Actinobacteria* and the decrease in *H. pylori* is a feature of the microbiota in patients with gastric cancer.^[^ ^62^ ^]^ At the species level, *Peptostreptococcus stomatis*, *Streptococcus anginosus*, *Parvimonas micra*, *Slackia exigua*, and *Dialister pneumosintesis* were enriched in gastric tumors and may play significant roles in the progression of gastric cancer.^[^ ^61^ ^]^

Because of the large number of bacteria in the large intestine, colorectal cancer is closely connected with the gut microbiota, which may affect the carcinogenesis and efficiency of treatments in several ways.^[^ ^9^, ^83^, ^84^, ^85^ ^]^ *Alistipes, Blautia, Pasteurellales*, and *Porphyromonas* are known to correlate with the clinical characteristics of patients with colorectal cancer.^[^ ^59^ ^]^ In a recent study, the abundance of some tumor‐associated bacteria such as *Fusobacterium, Bacteroides, Parvimonas, and Prevotella* is variable in different stages of colorectal cancer,^[^ ^63^, ^64^ ^]^ which demonstrates the effect of microbiota alteration on colorectal cancer progression. Among bacteria shown to have a connection to colorectal cancers, *Fusobacterium, Bacteroides*, *Selenomonas*, and *Prevotella* species exist in metastatic tumors, which suggests that these bacteria may be associated with metastasis of colorectal tumors.^[^ ^65^ ^]^ A study in 2021 showed that *E. coli* colonized in tumors may be beneficial to liver metastasis of colorectal cancer by disrupting the GVB and promoting the formation of a premetastatic niche.^[^ ^26^ ^]^ In addition, *B. fragilis* can recruit other bacteria to colorectal cancer sites and induce inflammatory reactions. *Bifidobacterium* was found to accumulate in tumor tissue during systemic administration and induce anti‐tumor immunity via STING signaling.^[^ ^18^ ^]^

Intratumor microbiota also exists in other types of cancer.^[^ ^15^, ^86^ ^]^ In ovarian and endometrial cancer, there are significant differences in the microbiome between normal and tumor tissues.^[^ ^66^ ^]^The abundance of *Chlamydia*, *Mycoplasma*, *Acinetobacter*, and *Brucella* increases in ovarian cancers^[^ ^66^ ^]^ while *Atopobium sp*. and *Porphyromonas sp*. are enriched in endometrial cancer and are of particular relevance to endometrial cancer.^[^ ^67^ ^]^ Nejman et al. reported that *Roseomonas mucosa*, *Sphingomonas US_602*, and *Staphylococcus cohnii* are the most common taxa in ovarian cancer tissues.^[^ ^15^ ^]^

Pancreatic cancer is a dangerous malignant tumor with a poor prognosis,^[^ ^87^ ^]^ and has attracted much attention. The existence of microbiota in pancreatic cancers has been proven in several studies.^[^ ^13^, ^15^, ^43^, ^49^, ^54^ ^]^ *Gammaproteobacteria* is considered the most common taxon of intratumor microbes in pancreatic cancers, which may influence anti‐tumor immunity and chemotherapy.^[^ ^54^ ^]^ Furthermore, a recent study showed that *Enterobacter asburiae, Klebsiella pneumoniae*, *Citrobacter freundii*, *F. nucleatum*, and *Enterobacter cloacae* are prevalent bacteria in pancreatic cancers, although their function is still uncertain.^[^ ^15^ ^]^ Other intratumor microbes such as *Malassezia globose*, *Pseudoxanthomonas*c, and *Saccharopolyspora* are also residents of pancreatic cancers.^[^ ^13^, ^43^ ^]^ Intratumor microbes play complex roles in pancreatic cancer progression. Some of the intratumor microbes such as *Pseudoxanthomonas* and *Streptomyces* may be beneficial to the long‐term survival of patients, although the mechanism is still unknown, while other taxa may promote the progression of cancers.^[^ ^49^ ^]^

Pancreatic cysts, a neoplasm that has a low risk of evolving into PDA, also have a specific microbiota. Members of *Bacteroides*, *Escherichia*, and *Acidaminococcus* are predominant in pancreatic cyst fluid.^[^ ^68^ ^]^ In another study, *F. nucleatum* and *Granulicatella adiacens* were found to be enriched in pancreatic cyst fluid.^[^ ^20^ ^]^ Importantly, these results suggest a possible conclusion that malignant transformation of benign tumors correlates with intratumor microbiota, although advanced evidence is still needed.

The skin is another organ with an abundant microbiome, which leads to the exposure of skin cancer to numerous microorganisms. In normal skin tissue, *Corynebacteria, Propionibacteria, and Staphylococci* are the three main genera of commensal microbiota, while the composition of microbiota is strongly connected with its location.^[^ ^69^ ^]^ In a recent study, bacteria in *Acinetobacter*, *Actinomyces*, *Corynebacterium*, *Enterobacter*, and *Streptococcus* genera were found in melanoma samples.^[^ ^7^ ^]^ Nejman et al. reported that *Paracoccus marcusii* and *Staphylococcus aureus* may be the most prevalent taxi in melanoma.^[^ ^15^ ^]^ For non‐melanoma skin cancers, *Staphylococcus aureus* is predominant in squamous cell carcinoma and is closely associated with carcinogenesis, while *Malassezia* is decreased in this cancer.^[^ ^70^ ^]^ The skin microbiota plays a complex role in tumorigenesis. A study using cultured cells showed that *Staphylococcus epidermidis* played a protective role against skin cancer via its production of 6‐N‐hydroxyaminopurine.^[^ ^71^ ^]^ However, in vitro studies are not sufficient to prove the function of microbiota and stronger evidence is required.

Breast cancer is the most commonly diagnosed cancer worldwide and is responsible for 6.9% of cancer‐related deaths.^[^ ^87^ ^]^ Although the breast is not a mucosal organ, it has been proven that there are abundant microbes present in breast tissues. *Bacillus, Pseudomonas*, and several other bacteria are inhabitants of the breast tissue.^[^ ^88^ ^]^ A study analyzing intratumor microbiota in seven types of tumors reported that microbiota in breast tumors exhibited the highest diversity among tumors analyzed in the research.^[^ ^15^ ^]^ *Streptococcus infantis*, *Lactobacillus iners*, *Corynebacterium US_1715*, and *F. nucleatum* were the most prevalent taxa in breast cancer. Compared with normal tissue, breast tumor tissue exhibits a significantly different composition of microbiota.^[^ ^72^, ^89^, ^90^ ^]^ A recent study compared the microbiome between tumor tissue and tumor‐adjacent normal tissue from individuals at high risk of breast cancer and tissue from healthy individuals.^[^ ^72^ ^]^ This research revealed that the abundance of *Pseudomonas*, *Porphyromonas*, *Proteus*, and *Azomonas* was relatively high at the genus level in tumor tissue, while some dominant genera in normal tissues such as *Propionbacterium* and *Staphylococcus* were hardly visible in tumor tissue, which may suggest the protective role played by these microbes. A recent study showed *that F. nucleatum* colonized breast cancer via the combination of Fap2 and Gal‐GalNAc, which promoted tumor growth and metastatic progression.^[^ ^25^ ^]^ Enterotoxigenic *B. fragilis* was also found to colonize breast tumors. Parida et al. revealed that enterotoxigenic *B. fragilis* induced the development of breast cancer by secreting *B. fragilis* toxin (BFT), which may play a role in mediating the *β*‐catenin and Notch1 axis.^[^ ^73^ ^]^ Researchers have also proposed the concept of “BFT” memory. In 2019, Tang et al. found that the accumulation of microbiome‐derived bile acids in breast tumors led to a good prognosis.^[^ ^91^ ^]^ However, whether these bile acids are produced by the intratumor microbiota or gut microbiota is uncertain.

A 2017 study demonstrated the existence of microbiota in the tumor tissue of head and neck squamous cell carcinoma samples.^[^ ^74^ ^]^ Researchers have mentioned the depletion of *Actinomyces* and the enrichment of *Parvimonas* at the phylum level in tumors relative to paired normal tissue, which may correlate with tumor progression. However, studies focusing on these cancers are still limited, and there is little evidence regarding the association between intratumor microbiota and these types of tumors.

## 5 Methods to Study Intratumor Microbiota

**Figure 5:**
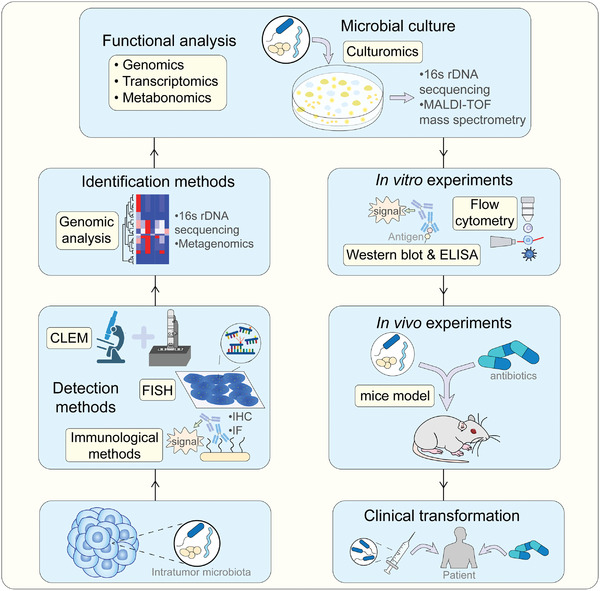
Method to study intratumor microbiota. Detection, identification, and functional analysis are necessary to focus for all research on intratumor microbiota. With the development of culture methods, in vitro and in vivo experiments may provide new insight into this field.

Although it is difficult to study the intratumor microbiota because of its low biomass, there are some methods used in studies of intratumor microbiota. Similar to researches on other microbial communities, fluorescence in situ hybridization (FISH), immunohistochemistry (IHC), immunofluorescence (IF), and other sensitive microbial detection methods were used that provides evidence for the existence of the intratumor microbiota. Beside detection of intratumor microorganisms, methods that can identify intratumor microorganisms are also of great importance. 16s rRNA gene amplicon sequencing and metagenomics are the most popular methods for identifying intratumor microbes. In addition, in order to study function characteristics of intratumor microorganisms, methods that help researchers gain pure culture of intratumor microorganisms is still needed. However, due to the low biomass and uncultivability of most intratumor microorganisms, methods that are independent from pure culture like metabolomics, genomics and transcriptomics are widely used to speculate potential functions and mechanisms of intratumor microbes. After that, researches focus on intratumor microbes should return to in vitro and in vivo experiments to verify previous results and provide insights for clinical translation. (**Figure** **5**).

### 5.1 Detection Methods

The low biomass of intratumor microbes leads to difficulties in definitively establishing their existence. Thus, essential microbial detection methods with low detection thresholds, especially in situ detection methods, are needed to enable these studies. FISH is a method that uses fluorescent DNA probes highly complementary to target sequences, which commonly use 16s rDNA as the target of probes in research focused on microbiota.^[^ ^92^, ^93^ ^]^ FISH has been used in studies focusing on the intratumor microbiota in several types of tumors, including breast cancer, lung cancer, and pancreatic cancer.^[^ ^13^, ^15^, ^55^, ^72^ ^]^ The main shortcoming of FISH is that the number of FISH probes that can be used in a sample is limited because of the fluorescence signal crosstalk. To solve this problem, different methods such as CLASI‐FISH and HiPR FISH have been developed and used to successfully analyze the gut microbiota.^[^ ^94^, ^95^ ^]^ Immunological technology also plays an important role in microbial imaging. Immunological technology uses specific antibodies to bind target molecules and uses antibodies labeled with enzymes (IHC) or fluorescein (IF) to identify these molecules. In recent studies, IF and IHC have been widely used to test and localize intratumor bacteria. For example, Nejman et al. used IHC with antibodies against bacterial lipopolysaccharide and lipoteichoic acid to detect intratumoral bacteria.^[^ ^15^ ^]^ However, due to superposition of colors and spectral overlap of the fluorophores, IHC and IF face difficulties in studying complex tissue. In 2017, a group of scientists developed a strategy for rapid multiplex IF using the exchange of fluorophore‐bearing DNA strands and DNA strands brought by specific antibodies, which can partly overcome the shortcomings of IF and may be used in analyzing cancer tissue with multiple microorganisms(*96*). Microscopic techniques are widely used to observe cells and molecules. Correlative light and electron microscopy (CLEM) is a powerful tool that combines the advantages of light microscopy and electron microscopy to accurately locate cells and molecules with high resolution.^[^ ^97^, ^98^ ^]^ Fluorescent probes are used in the CLEM technique to mark target molecules or cells. In studies on the intratumor microbiota, CLEM was used to locate bacteria.^[^ ^7^, ^15^ ^]^ For example, Nejman et al. demonstrated intracellular bacteria in breast tumors with CLEM.^[^ ^15^ ^]^ Compared with FISH and IF, CLEM allows researchers to observe the background of marked bacteria which and therefore obtain more information.^[^ ^97^ ^]^ In addition, with great progress in super‐resolution CLEM and intravital CLEM,^[^ ^99^, ^100^ ^]^ observing the behavior may be possible in the future. Therefore, CLEM may become more popular in studies of intratumor microbiota.

### 5.2 Identification Methods

Whether the focus of research is on environmental microbiomes, intestinal microbiomes, or intratumor microbiomes, the precise identification of microbiota using high throughput methods is of great importance. Traditional morphological identification methods based on morphological characteristics, biochemical characteristics, serum typing, and other characteristics can accurately identify several known strains and is still widely used in the identification of microorganisms. However, all those methods are based on pure cultures. Unfortunately, several types of intratumor microbes are uncultivated, which means traditional morphological identification methods are not feasible for the identification of those microorganisms. Thus, methods based on DNA sequencing are widely used in the identification of intratumor microbes because of their high accuracy and the relatively high availability of gene sequencing data. The 16s rRNA gene (rDNA) is a normal marker gene that exists in almost all living organisms, leading to the wide use of 16s rRNA sequencing, especially in the identification and evolutionary analysis of bacteria and archaea.^[^ ^101^, ^102^ ^]^ Another advantage of the 16s rRNA gene amplicon sequencing method is that there are both conserved regions that support the classification of organisms at the phylum level, and fast‐evolving regions that can be used to classify organisms at a finer taxonomic level.^[^ ^101^, ^102^ ^]^ Because of the universal application of this method, databases have amassed large quantities of sequence information of 16s rDNA, which further improves the usability of the method.^[^ ^101^, ^102^, ^103^ ^]^ Moreover, this technique is suitable for samples with low microbial content. Considering all the advantages, 16s rRNA gene amplicon sequencing is used in almost all experimental studies focusing on the intratumor microbiota in different tumors. Similar to other methods using marker genes, 16s rRNA gene amplicon sequencing is sensitive to the selection of primers.^[^ ^102^ ^]^ In a study focusing on intratumor microbiota in seven types of tumors, researchers amplified five short regions along the 16S rDNA and obtained higher resolution and coverage in species identification.^[^ ^15^ ^]^ However, the 16s rRNA gene amplicon can only identify known species, and its resolution can only reach the genus level at best with little functional information.^[^ ^101^ ^]^ Metagenomics is also important for the identification of intratumor microbes. Metagenomics is an untargeted sequencing method for all DNA in samples, including whole genome sequences of microbial communities, and is widely used in the analysis of complex microbiomes.^[^ ^104^ ^]^ Compared with marked gene sequencing, the resolution of metagenomics is higher, which can reach species and even the strain level.^[^ ^101^, ^105^, ^106^ ^]^ In addition, metagenomics can provide functional information. For example, a 2016 study analyzed 1003 reference genomes and identified them.^[^ ^106^ ^]^ The Cancer Genome Atlas (TCGA) is the main source of intratumoral microbiota that covers 33 types of cancers.^[^ ^107^ ^]^ Based on TCGA, intratumor microbes have been identified in several cancers.^[^ ^86^ ^]^ Recent studies have shown that the latest metagenomic data covers more types of cancers,^[^ ^108^ ^]^ which may lead to new progress in the field of intratumor microbiota. Furthermore, metagenomics can be used in combination with transcriptome analysis to eliminate interference caused by dead microorganisms and extracellular DNA.^[^ ^101^ ^]^ Despite the advantages mentioned above, there are some shortcomings of metagenomics, including higher cost, more complex operations, and inaccuracy caused by contamination or assembly.^[^ ^101^, ^109^ ^]^

In research on intratumor microbes, contamination caused by host DNA and DNA from environmental microbes is the biggest obstacle. Therefore, methods to discard untrusted data from TCGA need to be developed.^[^ ^110^ ^]^ In a study analyzing multiple types of cancers, researchers deleted 92.3% of the total sequence data to ensure the reliability of data in the analysis.^[^ ^86^ ^]^ In 2021, Dohlman et al. developed a decontamination algorithm that can remove contamination from TCGA data.^[^ ^111^ ^]^ As these methods develop, metagenomics can provide more powerful support for the research into intratumor microbiota.

### 5.3 Culture Methods

Metagenomics and other methods have provided a large amount of data about intratumor microbiota. However, pure culture of intratumor microorganisms is of great importance because results of in vitro and in vivo experiments based on the culture of those microorganisms are still needed. Several studies have shown that cultivation is a powerful supplement to metagenomics, with its essential contribution to describing new microbial species.^[^ ^112^ ^]^ Moreover, the acquisition of pure microbial culture is of great significance in further research enabling the functions and related mechanisms of these bacteria to be investigated. In recent years, great progress has been made in culturing microbes that are considered unculturable. The use of methods and conditions that mimic the natural environment is a common strategy for culturing microbes, and this approach led to the successful culture of *Candidatus Pelagibacter ubique* and many other microbes.^[^ ^113^ ^]^ Based on this strategy, several methods, such as membrane diffusion‐based cultivation and cultivation using microfluidic systems, have been developed and widely used in culturing environmental microbes.^[^ ^113^, ^114^ ^]^ Similarly, these methods may also be used for the culture of human microbiota. For example, Jalili‐Firoozinezhad et al. designed a microfluidic intestine‐on‐a‐chip and successfully cultured a large number of bacteria from 11 genera.^[^ ^115^ ^]^ In 2019, a group of researchers successfully purified and cultured human oral Saccharibacteria and SR1 bacteria.^[^ ^116^ ^]^ In this research, Cross and colleagues developed a method based on reverse genomics that can capture certain microbes by targeting specific protein epitopes. This method has shown its potential in separating and culturing microorganisms in any environment, which means that it can be used to culture intratumor microbes. Culturomics is another new technology that plays an important role in the research of human microbes. Culturomics uses multiple conditions to culture different types of microbes, which can then be identified using MALDI‐TOF mass spectrometry or 16s rDNA sequencing.^[^ ^113^ ^]^ This method can separate some uncultured or undetected microbes and determine the conditions for culturing these microbes. In the first study using culturomics, researchers identified several microbes from the gut for the first time and determined 70 useful culture conditions.^[^ ^117^ ^]^ In a 2016 study where culturomics was used, researchers identified 531 gut microbes that had never previously been identified in the gut, including 197 potential new species.^[^ ^118^ ^]^ A recent study using culturomics reported that gut bacteria gained new functions with changes in human lifestyle.^[^ ^119^ ^]^ The research also mentioned the potential association between industrialization and the high rate of horizontal gene transfer in the gut microbiota. Altogether, culturomics will provide more information about human microbiota because of its high‐throughput characteristics, it may also be used in the cultivation of intratumor microbes. However, because of the low biomass of intratumor microbes, there is no research reliable enough to separate and culture uncultured intratumor microbes successfully, which seriously limits related studies. On one hand, we believe that culture methods with a lower threshold are needed to solve this problem. On the other hand, we also need to know more about the interaction between intratumor microbes and the environment, including TMEs and other microbes, because the interaction may be the key factor affecting the success of culture techniques for intratumor microbes. Actually, the significance of those interactions has been proven in the culture of other microorganisms.^[^ ^120^ ^]^

### 5.4 Functional Analysis Methods

Species identification can only reveal the association between certain intratumor microbes and tumors, while the molecular mechanisms of these phenomena are uncertain. Considering the prevalence of uncultured bacteria in tumor tissue, omics techniques, including genomics, transcriptomics, and metabonomics, are used to analyze the mechanisms related to the development of cancers. In addition to identifying species, genomics can also be used to speculate on the potential functions and mechanisms of microbiota by identifying functional gene clusters. Transcriptomics can detect levels of different mRNA and provide information about the expression of different genes, which is also an important technique used in analyzing the functions and mechanisms of microorganisms because of its high‐throughput characteristics; however, it has some disadvantages including high cost and sensitivity to host RNA, especially rRNA contamination.^[^ ^101^ ^]^ In studies on intratumor microbiota, transcriptomics has been commonly used to analyze changes in human cells.^[^ ^25^, ^48^ ^]^ With the rapid development of cross‐species RNA‐seq,^[^ ^121^ ^]^ transcriptomics may reveal more mechanisms of interaction between tumors and tumor microbiota. Notably, a recent study partly overcame the difficulty of bacterial single‐cell RNA‐seq using a new method.^[^ ^122^ ^]^ Therefore, analysis of the interaction between tumor cells and intratumor bacteria at the single‐cell level may be possible in the future. Metabonomics is another important member of the omics techniques to analyze small‐molecule compounds in samples and provide metabolic characteristics of the sample. In research on microbiota, metabonomics provided much information about bacterial compounds related to cancers. In 2018, researchers described the metabolic profiles of the fecal metabolome using the metabonomics technique.^[^ ^123^ ^]^ Han et al. developed a metabolomics tool and reported the metabolic profiles of 178 gut microorganism strains.^[^ ^124^ ^]^ Mass spectrometry is a common technique used in metabolomics that can identify small‐molecule compounds by quality / composition ratio. In a study of intratumor bacteria in melanoma, researchers identified bacteria‐derived HLA peptides using mass spectrometry.^[^ ^7^ ^]^ Moreover, mass spectrometry imaging, as a new visualization technology of spatial metabolomics based on mass spectrometry, has been used in studies of host‐microorganism interactions.^[^ ^125^ ^]^ This also has great potential in studies of intratumor microbiota.^[^ ^26^ ^]^ Importantly, single omics techniques are not sufficient enough to support the deepening research in the field, and therefore the use of multi‐omics analysis will become more popular.

### 5.5 In Vivo and In Vitro Experiments

In addition to omics techniques, in vivo and in vitro experiments are essential to studies of intratumor microbiota. Traditional methods are commonly used at the in vitro level. Western blot, ELISA, and RNA‐seq are widely used to detect alterations in signaling pathways affected by intratumor microbiota. For example, Kong et analyzed the expression of CYP2J2, Keap1, and NRF2 with the technique of Western blot at the level of transcription and translation and therefore discovered that *F. nucleatum* can activate TLR4/Keap1/NRF2 axis and then promote the development of colorectal cancer.^[^ ^41^ ^]^ In addition, flow cytometry is one of the important techniques which can be used to analyze different types of cells, especially immune cells in co‐culture systems or isolated from tumor tissues. Researchers labeled different types of immune cells with antibodies and isolated them with flow cytometry in order to analyze the effects of intratumor microorganisms on the abundance of different immune cells.^[^ ^13^, ^37^, ^48^, ^49^ ^]^ At in vivo level, different types of tumor‐bearing mouse models are widely used to test the effects of gut microbiota and intratumor microbiota. Treatment with antibiotics is a common way to study the functions of intratumor microbiota.^[^ ^13^, ^37^, ^78^ ^]^ Antibiotic is always provided to mice in order to remove intestinal microorganisms by oral gavage while researchers also mix antibiotics in drinking water to administer. For mucosal sites, feeding bacteria is popular in studies focusing on gastrointestinal tumors.^[^ ^13^, ^26^ ^]^ In research completed by Jin and her colleagues, bacteria were inoculated into the lungs of mice by intratracheal administration after the original lung tumors.^[^ ^48^ ^]^ However, few in vivo studies have been conducted on non‐mucosal organs. In studies of these tumors, intravenous injection may be a good way to administer drugs and transplant microorganisms.^[^ ^25^ ^]^ Notably, studies focusing on the source of the intratumor microbiota may provide new insights into in vivo experiments. As for the detection of tumor samples from animal models, researchers used flow cytometry to isolate immune cells and other cells for analysis. In addition, they also detect the volume of tumors, levels of different cytokines in blood, and many other indexes to analyze the effects of intratumor microbiota on experimental animals.

## 6 Intratumor Microbiota and Antitumor Therapies

**Figure 6:**
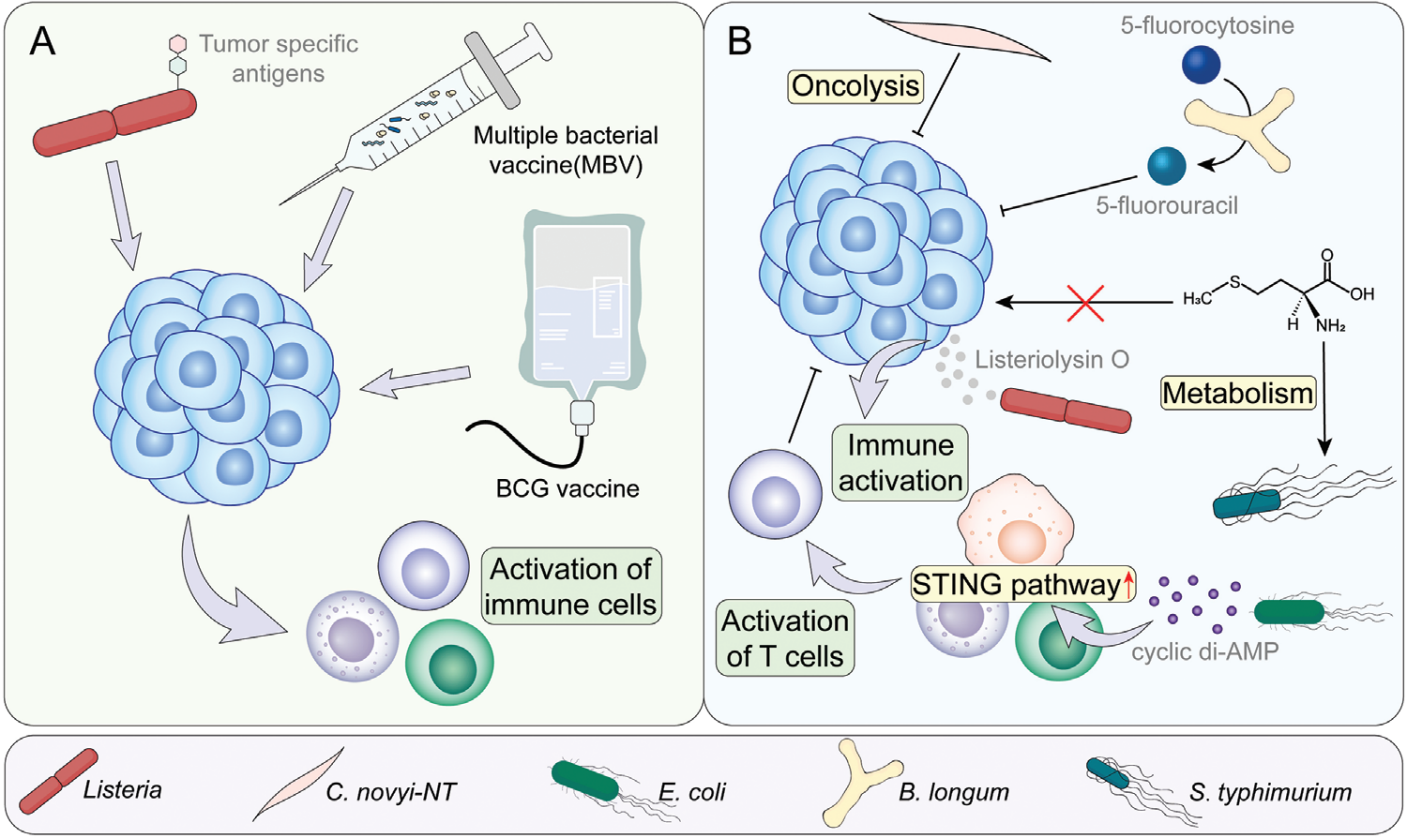
Clinical application of anti‐tumor bacterial treatment strategies. A) Biological agents inducing anti‐tumor immune response. This strategy uses dead or living bacteria to recruit active immune cells like CD8^+^ T cells and therefore initiates anti‐tumor immune response. B) Engineering bacteria inducing anti‐tumor response or used as carriers. Engineering bacteria can be modified to release certain products or conduct certain reactions to inhibit tumors. Also, engineering bacteria can also be used as carriers to carry toxin, immunostimulant, or other drugs.

With more understanding, diagnosis and treatment strategies based on intratumor microbiota can be developed.^[^ ^126^, ^127^ ^]^ In terms of diagnosis, researchers successfully distinguished tumor tissue from normal tissue via the composition of microbes in blood.^[^ ^86^ ^]^ As for therapies, two main strategies for microbial therapies have entered the clinical stages (**Figure** **6**): one strategy uses dead or living bacteria to activate anti‐tumor immune response through specific antigens. Bacillus Calmette‐Guerin(BCG) vaccine, multiple bacterial vaccines, and live, attenuated, double‐deleted Listeria monocytogenes are products of this idea. Another strategy is to use bacteria as carriers that can release toxins, immunostimulants, and other drugs.

In clinical therapies, treatment with BCG is effective for many cancers, especially bladder cancer.^[^ ^128^, ^129^ ^]^ Besides, certain bacteria such as Listeria and Salmonella typhimurium are used as delivery platforms because of their specific enrichment in tumors in order to better drug targeting.^[^ ^130^, ^131^, ^132^, ^133^, ^134^ ^]^ Furthermore, some researchers have developed novel drug delivery systems using outer membrane vesicles or artificial microbots.^[^ ^135^, ^136^ ^]^ Recently, a group of researchers designed an engineered E. coli that increased the concentration of L‐arginine and T cell infiltration in TMEs, thereby improving the anti‐tumor effect.^[^ ^137^ ^]^ Together, therapies based on microorganisms have attracted a lot of attention and some drugs have entered the clinical stage as shown in **Table** **2**. However, there are still a lot of difficulties on the way due to the relative lack of relevant studies. Although there are drugs such as YB1 that have gained great success in the treatment of canines, drugs such as VNP20009 and CRS‐207 did not yield satisfactory results, as they exhibited no anti‐tumor effect or did not inhibit bacterial infection. Therefore, in‐depth understanding of intratumor microbiota is of great importance.

**Table 2:** Clinical trials of anti‐tumor bacterial therapies

Bacterial species	Drug	Phase of trial	Indication	Identifier
multiple bacteria	Mixed Bacterial Vaccine (MBV)^[^ ^138^, ^139^ ^]^	Phase I	melanoma, sarcoma, gastrointestinal stromal tumor, head and neck cancer, transitional cell carcinoma, prostate cancer, ovarian carcinoma, esophageal cancer, breast cancer, renal clear cell carcinoma	NCT00623831
*Salmonella typhimurium*	VNP20009^[^ ^140^ ^]^	Phase I	solid tumor	NCT00006254, NCT00004216, NCT00004988
	SalpIL2^[^ ^126^ ^]^	Phase I	liver cancer, hepatoma, liver neoplasms, biliary cancer	NCT01099631
	SGN1	Phase I	advanced solid tumor	NCT05038150
	VXM01^[^ ^141^ ^]^	Phase I/II	glioblastoma, colorectal cancer, pancreatic cancer,	NCT02718443, NCT02718430, NCT01486329, NCT03750071
	YB1^[^ ^142^ ^]^	Pre‐clinical		
*Listeria*	JNJ‐64041809^[^ ^126^ ^]^	Phase I	prostatic neoplasms	NCT02625857
	JNJ‐64041757^[^ ^126^ ^]^	Phase I	non‐small‐cell Lung carcinoma,	NCT02592967
	ADU‐623^[^ ^126^ ^]^	Phase I	astrocytic tumors, glioblastoma multiforme, anaplastic astrocytoma, brain tumor	NCT01967758
	personalized live, attenuated, double‐deleted Listeria monocytogenes (pLADD)^[^ ^126^ ^]^	Phase I	colorectal neoplasms	NCT03189030
	CRS‐207^[^ ^126^, ^143^, ^144^, ^145^ ^]^	Phase II	Pancreatic Adenocarcinoma	NCT03006302, NCT03190265, NCT02243371, NCT02004262, NCT05014776, NCT01417000
	ADXS11‐001^[^ ^146^ ^]^	Phase II/III	head and neck cancer, anal cancer, rectal cancer, cervical cancer, non‐small‐cell lung carcinoma	NCT02002182, NCT02399813, NCT02853604, NCT02531854, NCT01266460
	ADXS31‐164^[^ ^147^ ^]^	Phase I/II	HER2 expressing solid tumors	NCT02386501
	ADXS‐NEO^[^ ^148^ ^]^	Phase I	colon cancer, head and neck cancer, non‐small cell lung cancer, urothelial carcinoma, Melanoma	NCT03265080
	ADXS‐503^[^ ^149^ ^]^	Phase I/II	non‐small cell lung cancer, squamous cell carcinoma, non‐squamous cell carcinoma	NCT03847519
	ADXS31‐142^[^ ^150^ ^]^	Phase I/II	prostate cancer	NCT02325557
*Escherichia coli*	SYNB1891	Phase I	solid neoplasm, lymphoma	NCT02718444
*Bifidobacterium longum*	APS001F^[^ ^126^ ^]^	Phase I/II	tumors	NCT01562626
*Clostridium novyi*	*Clostridium novyi*‐NT^[^ ^151^ ^]^	Phase I	Solid Tumor	NCT01924689, NCT03435952

## 7 Conclusions

As gut microbiota and its effect on tumors have attracted much attention, intratumor microbiota affecting tumorigenesis and cancer treatment is another interesting aspect of host‐microbiome interaction, which needs further investigation. In recent years, increasing evidence has proven that intratumor microbiota is widely present in different types of tumors and have complex functions.

With the deepening understanding of intratumor microbiota, mucosal organs and NATs are known as two potential sources of intratumor microbiota. In addition, as microorganisms were found in tumors occurring in non‐mucosal organs like breast and liver, circulatory system is considered a potential source of intratumor microbiota. For the mechanism, the interaction between lectin Fap2 expressed by *F. nucleatum* and Gal‐GalNAc expressed by tumor cells may cause the invasion of *F. nucleatum* while the mechanisms of how other intratumor microorganisms enter TMEs are still uncertain. In terms of action mechanisms, there are four major mechanisms by which the intratumor microbiota affects oncogenesis and cancer treatment. Part of intratumor microbes causes direct DNA damage which may lead to tumorigenesis. Some other intratumor microbes induce the activation of proinflammatory responses like the secretion of cytokines and the activation of NF‐kB pathway while other oncogenic pathways like TLR4/Keap1/NRF2 axis are also found to be activated by certain intratumor microbes. In addition, intratumor microbiota can induce immunosuppression through up‐regulating immune checkpoints and activating immunosuppressive cells while certain compositions of them can also enhance antitumor immunity. Besides, certain microorganisms like *Mycoplasma hyorhinis* and *Gammaproteobacteria* have the ability to metabolize certain antitumor drugs into an inactive form. Notably, the composition of the intratumor microbiota is highly heterogeneous in different types of tumors. In this review, we summarize the enriched taxa and reduced taxa of bacteria and fungi in different tumors. Low‐threshold techniques to detect and identify microbiota are needed to analyze the composition and functions of intratumor microbiota. Due to the low biomass of intratumor microbiota, culture of intratumor microbiota is difficult, which leads to limitations in in vitro and in vivo experiments. Here, we propose the potential use of culturomics and other culture methods in studies of intratumor microbiota, which may provide ideas to isolate intratumor microbiota and identify suitable conditions for culturing previously unknown microbes.

However, there are some obstacles to the study of intratumor microbiota. Environmental contamination seriously impacts the detection of intratumor microbiota and has to date made the presence of intratumor microbiota controversial. Thus, researchers have used a large number of negative controls to make the results reliable.^[^ ^15^ ^]^ Notably, metagenomic data are also influenced by environmental contamination, which makes these data inaccurate. Therefore, bioinformatic methods to discard untrusted data are of great importance in future research.

Based on the progression in the field of intratumor microbiota, microbial therapies show great application potential. At present, some microorganisms have been used as a killer of cancer cells, activator of antitumor immunity, or delivery platform of drugs. However, the clinical transformation of microbial therapy still faces plenty of problems. It is therefore important that the knowledge base with respect to intratumor microbiota is increased, which may, in turn, lead to surprising results with other bacterial treatment strategies in clinical trials.

In the future, we believe that the field of intratumor microbiota will attract more attention, with four aspects of this field that may become the focus of future research: 1) the development of decontamination algorithms for data analysis; 2) the source and colonization mechanisms of intratumor microbiota; 3) the culture of intratumor microbes and research into the underlying mechanisms; and 4) clinical transformation of tumor microbial studies.

In research on intratumor microbiota, contamination of samples is one of the main problems, which may cause the misjudgment of microbial species and functions. Moreover, because of the interference of environmental contamination, evidence to prove the existence of intratumor microorganisms is not strong enough, which makes the basis of relevant research questioned. In that case, besides improving the experimental design and setting more control groups, developing better decontamination algorithms for data analysis is also important. At present, decontamination of data, especially genomic data relies on manual operation of researchers, which is time‐consuming and laborious. Therefore, decontamination algorithms based on databases can help researchers to remove contamination of known microorganisms while those decontamination algorithms can also help researchers to detect new environmental microorganisms. Besides, we also hope that the use of decontamination algorithms can be extended to data from other omics which can provide more information about intratumor microbiota through a culture‐independent way.

With the development of technology, researchers discovered microorganisms in several types of cancers. However, we know little about the source and colonization mechanisms of those microorganisms. Dyes for labeling living microorganisms can help researchers observe the progression that microorganisms invade TMEs, which may help researchers to solve the problem. In addition, in‐depth understanding of environmental preference of microorganisms and their behaviors in their original habitats may also provide prompts to researchers. Comparing the composition of microorganisms in potential sources and TMEs may help researchers to determine the exact source of intratumor microbiota in different tumors. Further, enriched pathways related to microorganisms in different tumors may also provide new insights into colonization mechanisms of intratumor microbiota. Therefore, bioinformatics analysis of those pathways is still needed.

As mentioned in the article, though omics technology has made great progress, culture of intratumor microorganisms is still important. Culturology has been used in the research of environmental microorganisms and can also be used to study intratumor microorganisms that are difficult to culture. Researchers can also form culture conditions similar to the original environment of intratumor microorganisms to isolate and culture them. Also, some other methods used to isolate and culture environmental microorganisms mentioned in this article can also help researchers focus on intratumor microbiota. In future studies, culturology based on a medium containing different components may make it possible to cultivate some intratumor microbiota. Considering the interaction network of different microorganisms, as well as tumor cells and immune cells, co‐culture of intratumor microorganisms and those cells can help researchers to verify the functions and mechanisms of intratumor microbiota in in vitro and in vivo experiments. Besides, determination of microbial culture conditions can in turn provide hints for the study of mechanisms. For example, medium composition and environments of culture may suggest key factors necessary for the colonization of intratumor microbiota while the co‐culture of those microorganisms and different cells may reveal the effect mechanisms of intratumor microbiota.

**Figure 7:**
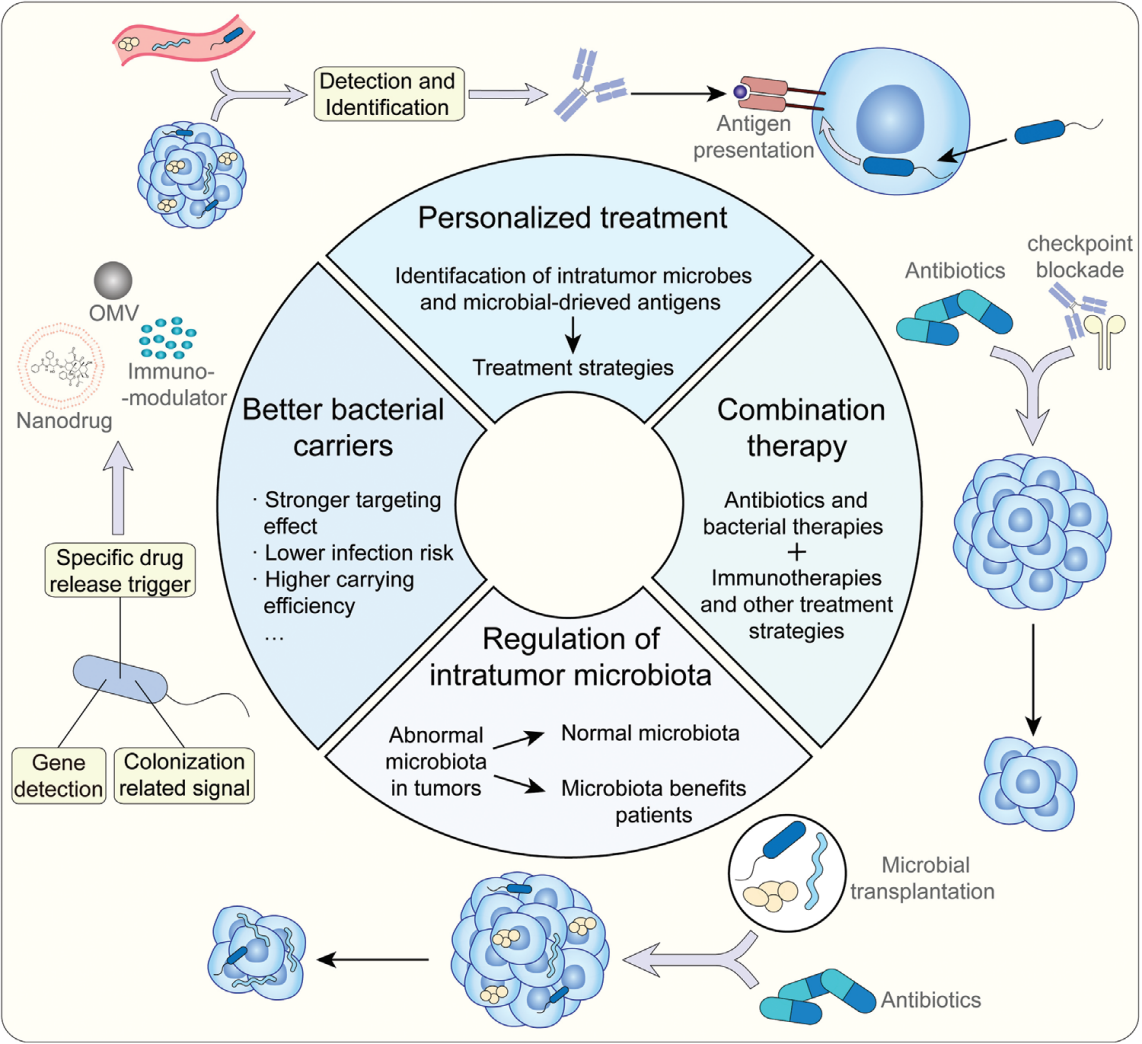
Prospect of clinical application based on intratumor microbiota. Personalized therapies will be beneficial because of the heterogeneity of intratumor microbiota. Antibiotics and bacterial therapies combined with other anti‐tumor therapies may improve results. Normalizing intratumor microbiota and transplanting certain microorganisms are also potential strategies to enhance the efficiency of anti‐tumor therapies.

As for clinical transformation, four directions may have a higher fever in the future (**Figure** **7**). Considering the heterogeneity of the intratumor microbiota, personalized treatment strategies are attractive because of their high efficiency and targeting effect. Researchers have successfully diagnosed cancers with microorganisms in the blood.^[^ ^86^ ^]^ In addition, bacteria‐derived peptides have been found in melanoma,^[^ ^7^ ^]^ which can be used as potential anti‐tumor targets. Hence, the development of detection and identification technologies will enable the development of personalized treatments with antibodies or other biologics based on personal intratumor microbiota. Like other anti‐tumor therapies, bacterial therapies and antibiotics can also be used in combination with other therapies, such as immunotherapy and chemotherapy. The combination of engineering bacteria and using a checkpoint blockade approach has entered clinical trial stages, while antibiotics have also been used as adjuvant drugs in some clinical trials. Normalizing tumor immunity is an important concept in immunotherapy. In the clinical transformation of intratumor microbiota, normalizing the intratumor microbiota is also a potential way to improve the condition of patients. Moreover, with the achievement of fecal microbiome transplantation and research revealing microorganisms related to the prognosis of pancreatic cancers,^[^ ^49^ ^]^ transplanting microorganisms into TMEs may bring surprising results, although related research is limited. Exploration of mechanisms affecting colonization and the function of intratumor microbiota, bacterial carriers will be more targeted with lower infection risk and higher carrying capacity. Thus, intratumor microbiota as an important component of the TME, will be a focus area for tumor research and for the development of new targets for anti‐tumor treatment.

In summary, intratumor microbiota is an important and heterogeneous component of TMEs, which play complex roles in the occurrence and development of tumors. There are still many problems to be solved in this field, which need the development of technology and hard work. Notable, researches on intratumor microbiota have strong clinical transformation potential, which may be the next hotspot of antitumor therapy.

## Conflict of Interest

The authors declare no conflict of interest.

## Author Contributions

Y.X., F.X., X.Z., and L.Z.(Lei Zhang) contributed equally to this work. Y.X., F.X., X.Z., and L.Z.(Lei Zhang) conceived and drafted the manuscript, drew the figures and summarized the tables. Y.X., F.X., X.Z., L.Z.(Lei Zhang), F.W., J.H., H.Y., L.Z.(Linghui Zeng), and F.Z. discussed the concepts of the manuscript. L.Z.(Long Zhang) approved the version to be submitted.
